# Selective Oxidation of Ethane to Acetic Acid Catalyzed by a C-Scorpionate Iron(II) Complex: A Homogeneous vs. Heterogeneous Comparison

**DOI:** 10.3390/molecules25235642

**Published:** 2020-11-30

**Authors:** Inês A. S. Matias, Ana P. C. Ribeiro, Luísa M. D. R. S. Martins

**Affiliations:** Centro de Química Estrutural, Departamento de Engenharia Química, Instituto Superior Técnico, Universidade de Lisboa, 1049-001 Lisboa, Portugal; ines.matias@tecnico.ulisboa.pt

**Keywords:** ethane oxidation, acetic acid, magnetic catalyst, core-shell, C-scorpionate, iron, heterogeneous catalysis, green metrics

## Abstract

The direct, one-pot oxidation of ethane to acetic acid was, for the first time, performed using a C-scorpionate complex anchored onto a magnetic core-shell support, the Fe_3_O_4_/TiO_2_/[FeCl_2_{κ^3^-HC(pz)_3_}] composite. This catalytic system, where the magnetic catalyst is easily recovered and reused, is highly selective to the acetic acid synthesis. The performed green metrics calculations highlight the “greeness” of the new ethane oxidation procedure.

## 1. Introduction

**Figure 1:**
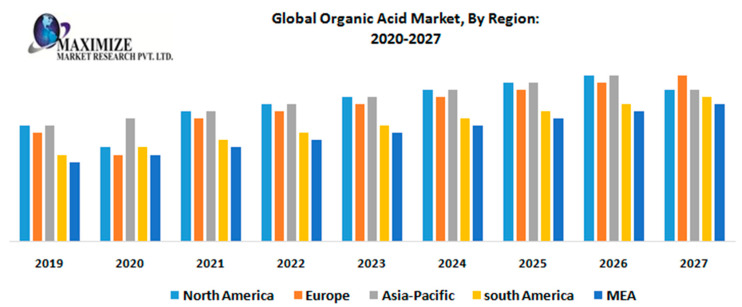
Worldwide acetic acid market forecasts. Reproduced with the permission of [2].

High-value, sustainable commodities obtained through novel catalytic processes are the answer towards developing a cleaner, more efficient, modern and future oriented industry. A significant example is the production of acetic acid, a large tonnage commodity of high importance in view of its wide industrial use and word demand (the global acetic acid market reached a volume of 17.28 million tons in 2019 [1]), which is expected [2] to continue increasing in the next few years (Figure 1).

**Scheme 1:**
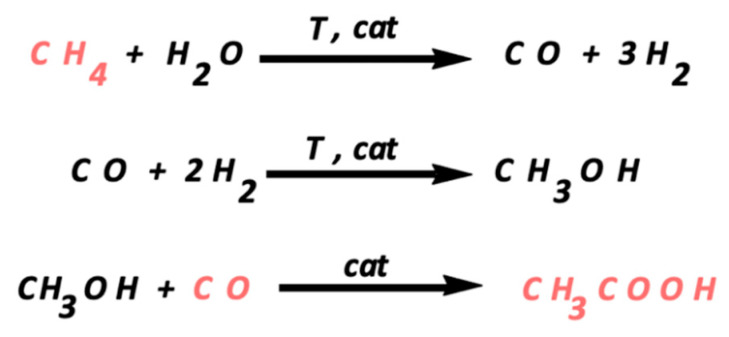
Acetic acid production from the raw materials, involving the carbonylation of methanol in the third step.

The current preferred manufacturing of acetic acid is through carbonylation of methanol. This process uses methanol and carbon monoxide as raw materials, which are produced from natural gas, coal and heavy residual oil, among other materials [3] (Scheme 1).

In 1960, BASF (using cobalt catalysts) industrialized the aforementioned acetic acid manufacturing process, followed by Monsanto (with rhodium catalysts) in 1970, and in 1996 a new process for the carbonylation of methanol to acetic acid was announced by BP Chemicals, based on a promoted iridium catalyst package named Cativa^TM^. Although the most recent process presents benefits such as the high yield of acetic acid, the disadvantages include environmental ones and the risk of severe corrosion of the equipment (resulting from the required iodide cocatalyst). The needed alternative of synthesizing acetic acid directly through oxidation of a gaseous alkane [4,5] that appears to be much wiser, clean and cheaper, is also challenging.

Ethane is the most suitable alkane for such a process by choice, although some advances on obtaining acetic acid from methane (via carboxylation with CO) have also been reported [6,7,8,9]. In 2005, SABIC of Saudi Arabia commercialized an acetic acid plant [10] based on a low-cost ethane oxidation process where the catalyst is a calcined mixture of oxides of Mo, V, Nb and Pd, but with selectivities to acetic acid of just 80%.

A wide range of catalysts have been tested for the partial oxidation of ethane to acetic acid, either in homogeneous or heterogeneous conditions [11]. However, so far, none has demonstrated the performance required for industrial commercialization.

**Figure 2:**
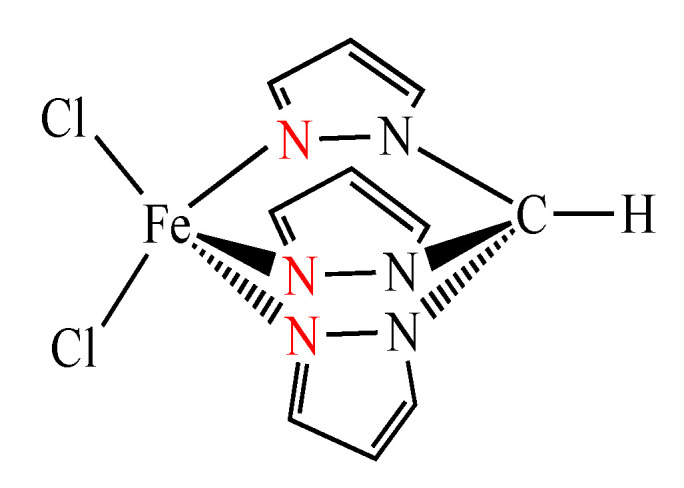
Structure of the dichloro hydrotris(pyrazol-1-yl)methane iron(II) complex [FeCl_2_{κ^3^-HC(pz)_3_}] (pz = pyrazol-1-yl).

In pursuit of our interest on oxidation catalysis of industrial significance [5,12,13], herein, we successfully explored the catalytic activity of the C-scorpionate iron(II) complex [FeCl_2_{κ^3^-HC(pz)_3_}] (pz = pyrazol-1yl, Figure 2) [14] for the selective oxidation of ethane to acetic acid. This iron compound previously proved its efficiency in the catalytic oxidation of cyclohexane [15,16,17,18,19,20,21,22,23,24] but was not applied for other (more challenging) alkane substrates. Moreover, to our knowledge, no iron(II) coordination compound was, to date, reported as a catalyst for the oxidation of ethane to acetic acid.

C-homoscorpionates are tripodal nitrogen tris(pyrazol-1-yl)-methane ligands (introduced [25] by Trofimenko in the late 1960s), the designation of which arises from the peculiar coordination mode of these chelators to a metal like a scorpion attack, pictorially compared to an embrace of the metal center, like the pincers and tail of a scorpion catching and stinging its pray. Their easy interchange of coordination modes between tri- and bi-dentate is believed to be responsible for the structural versatility and chemical (catalytic) performance of their metal complexes.

Very recently, a hybrid material consisting of the aforementioned complex [FeCl_2_{κ^3^-HC(pz)_3_}] immobilized at magnetic Fe_3_O_4_/TiO_2_ core-shell particles, Fe_3_O_4_/TiO_2_/[FeCl_2_{κ^3^-HC(pz)_3_}], was successfully prepared [26] and used as a catalyst for the selective oxidation of 1-phenylethanol to acetophenone. The magnetic, oxide covered, Fe_3_O_4_/TiO_2_ core-shell was revealed to be a convenient support for the iron complex, enabling the composite to be collected by an external magnetic field [27]. This possibility is a significant operational advantage, as the usually required separation techniques (e.g., filtration or centrifugation), leading to high catalyst losses, are avoided.

Despite the current significant interest towards the by far more challenging oxidation of ethane to acetic acid, there are relatively few reports regarding the low temperature, heterogeneously catalyzed selective oxidation of ethane [11], specially using non-precious metals as catalysts. Fe/ZSM-5 (30) is the so far unique iron-based catalyst which led to productivities of up to 65 mol ethane converted·kg_cat_^−1^·h^−1^ at 56% ethane conversion, with acetic acid being the major product (70% selectivity, 39.1% yield) [11].

In this work, the selective oxidation of ethane to acetic acid, successfully catalyzed by the reusable magnetic Fe_3_O_4_/TiO_2_/[FeCl_2_{κ^3^-HC(pz)_3_}] composite, is presented. To the best of our knowledge, this is the first time that a magnetic core-shell iron composite [or even an iron(II) coordination compound] has been used as a catalyst for the direct one-pot oxidation of ethane to acetic acid.

## 2. Results and Discussion

**Figure 3:**
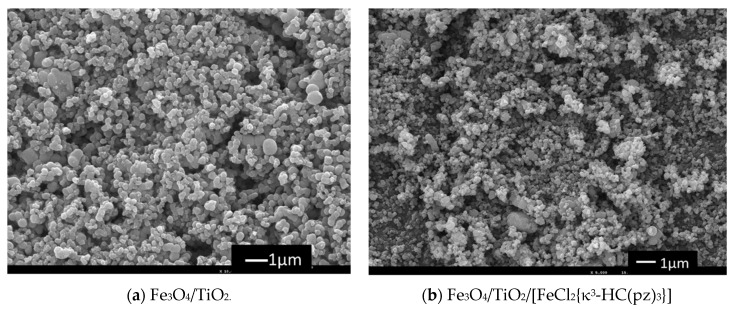
Scanning electron microscopy images of core-shell Fe_3_O_4_/TiO_2_ (**a**) and of Fe_3_O_4_/TiO_2_/[FeCl_2_{κ^3^-HC(pz)_3_}] composite (**b**).

The freshly synthesized [14] dichloro hydrotris(pyrazol-1-yl)methane iron(II) complex [FeCl_2_{κ^3^-HC(pz)_3_}] (pz = pyrazol-1-yl) was immobilized onto the above Fe_3_O_4_/TiO_2_ magnetic core-shells using our very recently reported procedure [26] (see materials and methods section) and the resulting Fe_3_O_4_/TiO_2_/[FeCl_2_{κ^3^-HC(pz)_3_}] magnetic composite was analyzed by using the usual spectroscopic techniques such as scanning electron microscopy and energy dispersive X-ray (SEM/EDX), thermogravimetry-differential scanning calorimetry (TG-DSC) and X-ray powder diffraction (XRD). The spectroscopic results fully matched the ones that were previously obtained [26]. For example, the structure and morphology of Fe_3_O_4_/TiO_2_/[FeCl_2_{κ^3^-HC(pz)_3_}] composite were assessed by using SEM/EDX (Figure 3). The magnetite-rich powder coated by TiO_2_ (Fe_3_O_4_/TiO_2_) with a non-spherical morphology is clearly observed in Figure 3a, where TiO_2_, as expected, covers the entire surface. The Fe_3_O_4_/TiO_2_/[FeCl_2_{κ^3^-HC(pz)_3_}] composite (Figure 3b) exhibits also a non-spherical morphology and the correspondent EDX analysis detected the presence of the C-scorpionate iron(II) complex. An iron content of 0.3%wt was determined by using inductively coupled plasma atomic emission spectroscopy.

### 2.1. Oxidation of Ethane Catalysed by [FeCl_2_{κ^3^-HC(pz)_3_}]

In order to assess the catalytic performance of the C-scorpionate Fe(II) complex [FeCl_2_{κ^3^-HC(pz)_3_}] for the direct one-pot oxidation of ethane to acetic acid, the reaction was performed using potassium peroxodisulfate as an oxidant and the iron complex (ethane/Fe ratio: 67) as a catalyst.

The catalytic experiments were initiated by choosing the traditional solvent for the oxidation of ethane, i.e., trifluoroacetic acid (TFA) [28,29,30,31,32]. A maximum acetic acid yield of 13.6% was attained after 20 h at 80 °C under a C_2_H_6_ pressure of 3 atm (entry 2, Table 1) with a remarkable selectivity (acetic acid was the only product detected by using chromatographic analysis).

**Figure 4:**
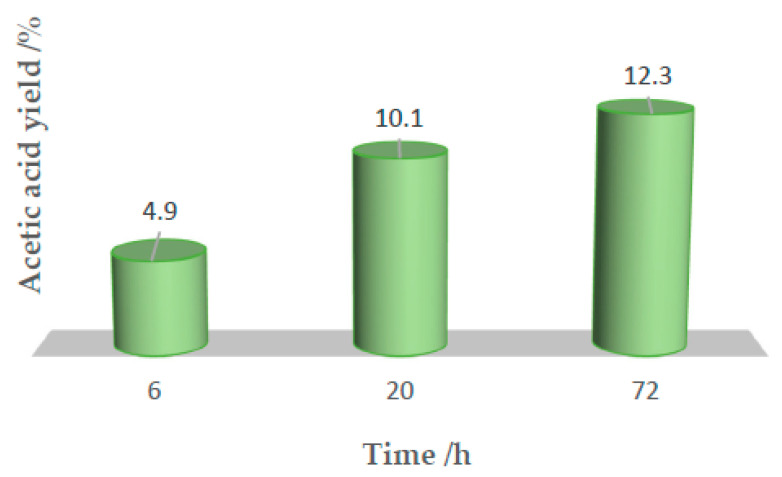
Acetic acid yield evolution over reaction time in a water/acetonitrile mixture.

Then, a mixture of water/acetonitrile (1:1) was chosen to replace TFA as a solvent, due to the hazardous character of the later, and the reactions were conducted exactly under the same reaction conditions. In aqueous acetonitrile at 80 °C for 20 h, 10.1% of ethane was converted into acetic acid (entry 5, Table 1) as the only reaction product. An increase in the reaction time (until 72 h) led to only a slight increase of acetic acid yield to 12.3% (entry 6, Table 1), as depicted in Figure 4.

The use of FeCl_2_.4H_2_O as catalyst produced approximately 4% of yield, after 20 h. This is an indication that iron species are active but the scorpionate complex is much more efficient that the iron salt (compare entries 5 and 13 of Table 1). This suggests the favorable involvement of the C-scorpionate ligand in the iron assisted steps of this catalytic oxidation reaction. Moreover, the easy interchange between tri- and bi-dentate coordination modes of the C-scorpionate ligand provides the required structural and chemical versatility to the iron complex as a catalyst for the oxidation of ethane.

**Figure 5:**
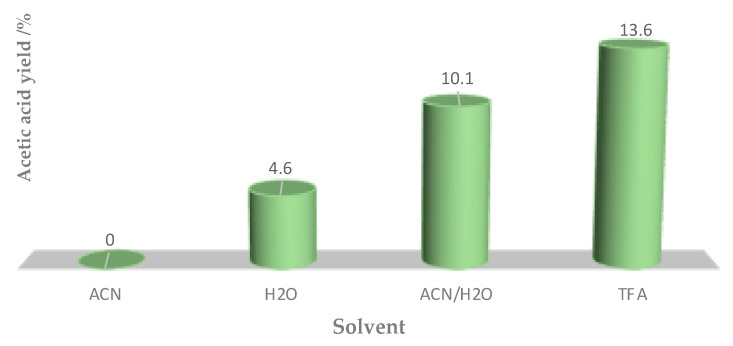
Acetic acid yield evolution in different solvents (0.04 mmol of [FeCl_2_{κ^3^-HC(pz)_3_]; 20 h at 80 °C).

The type of solvent had a crucial effect (Figure 5) on the conversion of ethane under the tested reaction conditions (compare entries 2, 5, 7 and 8 of Table 1). The inhibiting effect of acetonitrile on the formation of acetic acid was minimized by the addition of water, while TFA allowed us to achieve the highest oxidation yield. The observed effect could be explained by the participation of water in the active site turnover, namely by filling surface oxygen vacancies to oxidative dehydrogenation of surface hydroxy groups. It is still not clear such effects are due to a direct participation of water in the main reaction pathway [33] or to a specific interaction of H_2_O with certain catalyst.

The knowledge that Shul’pin and co-workers previously undertook [34] the partial oxidation of ethane over NaVO_3_ + H_2_SO_4_ and H_2_O_2_ in acetonitrile achieving the reaction selectivity trend: ethanol (51% selectivity), acetaldehyde (32%) and acetic acid (17%), prompted us to test the acid effect on our catalytic system. It was also found that the presence of an acidic medium resulted in an inhibiting effect on the oxidation of ethane (entry 11, Table 1).

Our C-scorpionate Fe(II) complex [FeCl_2_{κ^3^-HC(pz)_3_}] exhibited much higher selectivity to the formation of acetic acid than the previously tested iron(III) complexes. In fact, the partial oxidation of ethane, using [Fe^III^(Me_3_tacn)(Cl-acac)Cl]^+^ type catalysts and oxone (KHSO_5_) as oxidants, reported by Tse et al. [35] led to a C_2_ oxygenate selectivity of 80% of acetic acid and 20% of ethanol. The authors have also studied ligand effects by using different bidentate and tridentate ligands to stabilize the active site. The most active catalyst was the B-scorpionate [Fe^III^(Tp)_2_][ClO_4_] [Tp = hydrotris(1-pyrazolyl1)-borate] that showed a TOF of 12.0 mol ethane converted mol catalyst^−1^·h^−1^ at room temperature.

In spite of the herein revealed excellent selectivity to acetic acid formation (although in modest yields), one main disadvantage of the [FeCl_2_{κ^3^-HC(pz)_3_}] catalyst was its lack of reusability. In fact, its high solubility in aqueous acetonitrile impaired its easy recovering and possible reusing.

Thus, our next goal was to try to stabilize the C-scorpionate Fe(II) complex by anchoring it at an inert (and easy to recover) supporting material as the magnetic Fe_3_O_4_/TiO_2_ core-shells. The Fe_3_O_4_/TiO_2_/[FeCl_2_{κ^3^-HC(pz)_3_}] composite was successfully achieved (see above) and its catalytic activity for the oxidation of ethane to acetic acid was studied, as described in the following section.

### 2.2. Oxidation of Ethane Catalysed by the Magnetic Fe_3_O_4_/TiO_2_/[FeCl_2_{κ^3^-HC(pz)_3_}] Composite

**Figure 6:**
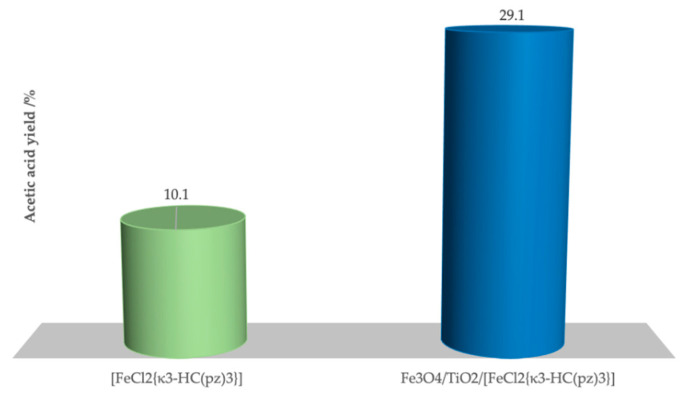
Acetic acid yield obtained by homogeneous (**left**) and heterogeneous (**right**) oxidation of ethane at 80 °C in a water/acetonitrile mixture.

The oxidation of ethane was undertaken in the presence of the prepared magnetic Fe_3_O_4_/TiO_2_/[FeCl_2_{κ^3^-HC(pz)_3_}] composite. A significant enhancement of the catalytic activity of the C-scorpionate iron(II) complex was achieved (Figure 6) by its immobilization at the magnetic Fe_3_O_4_/TiO_2_ core-shell (which did not exhibit catalytic activity for this reaction per se; see entry 17, Table 2). A maximum yield of 29.1% (entry 4, Table 2) of acetic acid was reached under the same conditions as the conditions previously used for [FeCl_2_{κ^3^-HC(pz)_3_}] (which led to a maximum yield of 10.1%, Table 1), while maintaining the selectivity of the homogeneous system.

It is worth highlighting that high yields (up to 21.3%, entry 5, Table 2) were also obtained with half of the [FeCl_2_{κ^3^-HC(pz)_3_}] amount used in the homogeneous experiments.

The previously observed (Section 2.1) inhibiting effect of an acidic medium on the homogeneous oxidation of ethane was also observed for the oxidation catalyzed by the Fe_3_O_4_/TiO_2_/[FeCl_2_{κ^3^-HC(pz)_3_}] composite (entry 7, Table 2). Moreover, the presence of a base (NaOH) as additive also resulted in the inhibition of the iron composite catalytic activity (entry 8, Table 2).

**Figure 7:**
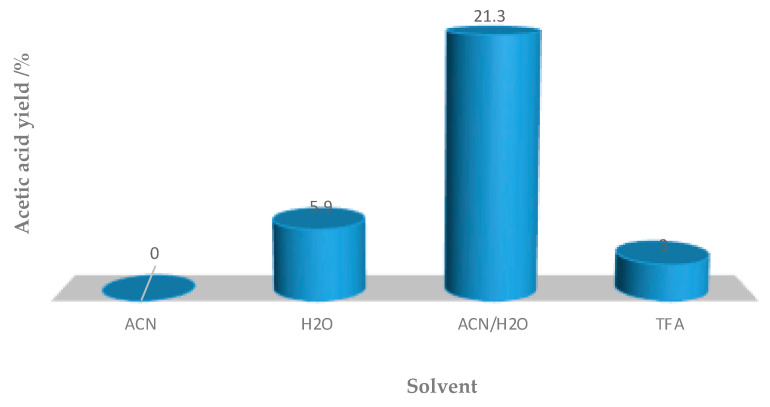
Acetic acid yield evolution in different solvents (0.02 mmol Fe_3_O_4_/TiO_2_/[FeCl_2_{κ^3^-HC(pz)_3_}], 20 h at 80 °C).

Interestingly, the solvent effect on the heterogeneous system differed from the effect found under homogeneous conditions (compare Figure 5 and Figure 7). The Fe_3_O_4_/TiO_2_/[FeCl_2_{κ^3^-HC(pz)_3_}] composite exhibited a significantly superior performance in the (1:1) water/acetonitrile mixture (up to 21.3% yield of acetic acid) than in the hazardous TFA (3% acetic acid yield, entry 1, Table 2). This decrease is probably due to the fact that TFA can be chemisorbed on the surface of the TiO_2_ films as a trifluoroacetate complex. This TFA complex bound on the surface of TiO_2_ can act as an electron scavenger and, thus, reduces the recombination of electrons and holes. The TFA eventually decomposes under the strong oxidizing environment of the catalytic process.

**Figure 8:**
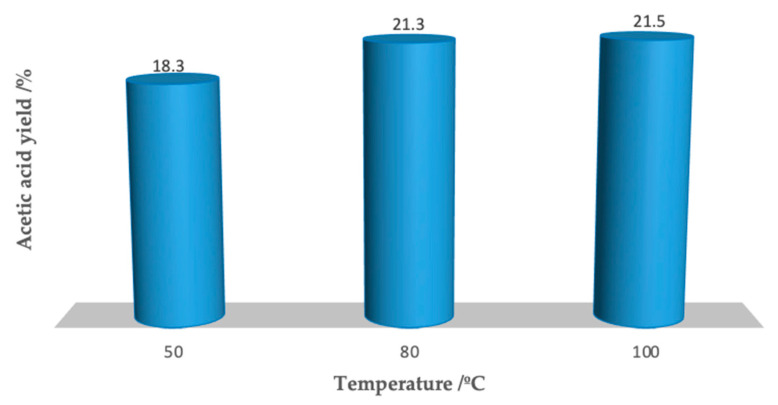
Effect of the temperature on the acetic acid yield evolution (0.02 mmol Fe_3_O_4_/TiO_2_/[FeCl_2_{κ^3^-HC(pz)_3_}], 20 h at 80 °C).

The temperature plays an important role in the oxidation of ethane in the presence of Fe_3_O_4_/TiO_2_/[FeCl_2_{κ^3^-HC(pz)_3_}], conceivably due to the high energy involved in the activation of such a small reactive substrate such as ethane (Figure 8). Although this does not occur as significantly as under homogeneous conditions (see entries 10 and 5, respectively, of Table 1), the product yield increased under heterogeneous as the temperature rose from 50 to 80 °C (entries 9 and 5, respectively, Table 2), whereas for higher temperatures (100 °C), it remained almost unchanged (compare entries 5 and 10 of Table 2). Therefore, like under homogeneous conditions, the temperature of 80 °C was considered to be the most adequate for heterogeneous conditions, combining yield and energy efficiency.

Another source of iron (II), Fe_3_O_4_/TiO_2_/FeCl_2._2H_2_O, was tested (entries 16 and 17, Table 2) and it is clear that, as observed under homogeneous conditions (see above), the [FeCl_2_{κ^3^-HC(pz)_3_}] scorpionate complex is more active as a catalyst for the oxidation of ethane than the iron in the tested salt (compare entries 5 and 17, Table 2). In addition, the Fe_3_O_4_/TiO_2_ core shell was tested as a catalyst under the same reaction conditions but no catalytic activity towards the oxidation products formation was detected. This is an indication that the role of the core shell as a support is efficient due to its high stability.

**Figure 9:**
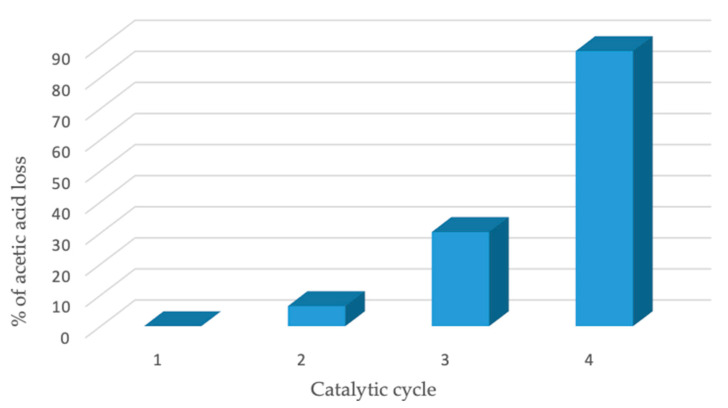
Recycling tests for the oxidation of ethane catalyzed by Fe_3_O_4_/TiO_2_/[FeCl_2_{κ^3^-HC(pz)_3_}]. % of acetic acid loss = (yield of n cycle per initial yield) × 100.

The activity of Fe_3_O_4_/TiO_2_/[FeCl_2_{κ^3^-HC(pz)_3_}] composite in the successive oxidative reactions was evaluated through its potential recyclability for up to four consecutive catalytic cycles, as described in Section 3.2, using the optimized conditions (entry 4 of Table 2). As expected, the recovering of the magnetic catalyst was extremely easy and efficient, avoiding catalyst loss during the separation process. As presented in Figure 9, under the above-mentioned reaction conditions, there was a small initial decrease of 6.5% in the catalytic activity between 1st and the 2nd cycle. Then, from the 2nd to the 3rd cycle, the acetic acid production loss increased by up to 30% (indicating a higher loss of activity) and by the end of the 4th cycle, the loss was 89%. This consecutive decreasing activity was sufficient to prevent further catalytic cycles from being run.

**Figure 10:**
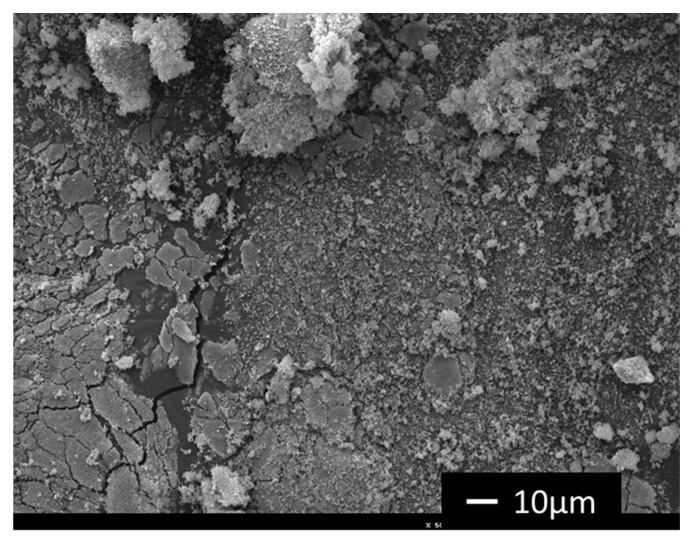
A scanning electron microscopy (SEM) image of magnetic Fe_3_O_4_/TiO_2_/[FeCl_2_{κ^3^-HC(pz)_3_}] composite after the 4th reaction cycle.

Although the % of acetic acid loss, which is the ratio between the yield of *n* cycle over the yield obtained in the first cycle shows a continuous increase (Figure 9), the selectivity of 100% to acetic acid was always observed. This could mean that the catalyst suffered a deactivation and a partial leaching process occurred. SEM/EDX analysis of Fe_3_O_4_/TiO_2_/[FeCl_2_{κ^3^-HC(pz)_3_}] after the 4th consecutive cycle (Figure 10) revealed the occurrence of leaching of the C-scorpionate iron(II) complex from the core-shell surface, in accordance with the diminishing of the composite catalytic activity.

Despite the current high interest towards the activation of lower alkanes, namely by its activation, there are still relatively few reports regarding heterogeneously catalyzed selective oxidation of ethane. Nevertheless, our magnetic catalytic system was benchmarked to other heterogeneous catalytic systems that were previously reported, and we can essentially highlight that: (i) Li et al. [36] used a non-aqueous environment system (methylnitrate and trifluoroacetic acid), where acetic acid and formic acid are formed after 12 h at 85 °C. The attained yield and acid selectivity values were lower than in our case; (ii) Shul’pin et al. used titanosilicalite TS-1 to catalyze the partial oxidation of ethane with H_2_O_2_ but did not produce acetic acid [37]; (iii) Rahman et al. showed the direct oxidation of ethane to acetic and formic acid using ZSM-5, aqueous H_2_O_2_, 30 bar of ethane, 120 °C, 2 h and PPh_3_ as the relevant variables. Under these conditions, 35.1% ethane conversion with major product selectivities of acetic acid (48.5%), formic acid (36.3%) and CO_2_ (11.9%) were reached [38], which again were lower acetic acid yield values than the ones reported herein; and iv) iron phthalocyanine complexes [39] supported at SiO_2_ ((FePc)_2_N/SiO_2_) led to higher reaction yields (34%) but lower selectivity (69%), under mild conditions (60 °C).

### 2.3. Green Metrics

To assess the quantitative improvement brought about by our ethane oxidation procedure, several green analytical chemistry metrics [40] were calculated and are presented in Table 3.

The use of the mixture water/acetonitrile led always to lower E-factor values than the ones obtained when using TFA as solvent (compare entry 1 and 2 or 3 and 4 of Table 3), indicating a lesser amount of waste is generated and, therefore, that the process exhibits higher environmental sustainability. On the other hand, an atom economy (EA) of 20% (Table 3) is a clear indication that one of the species is off balancing the atomic balance of the process: the oxidant, K_2_S_2_O_8_ (reagent to oxidant ratio of 1:6). *AU* values (Table 3) also reflect the “green economy” of the process, as all components of the reaction were used to produce the desired acetic acid, minimizing waste production. The Solvent and Catalyst Environmental Impact Parameter should have a value as low as possible if only if all materials used in the process are recycled, recovered or eliminated; otherwise, it will measure the need for improvement in the process. It is clear that a simple change in the solvent can lead to a decrease of approximately 80% in the value of *f* (entry 3 and 4 of Table 3) for the heterogeneous catalytic process. In the homogeneous process, the improvement in the metrics is 85% just by changing the solvent (entry 1 and 2 of Table 3). Therefore, it is of crucial importance to assess the improvement in the processes by using metrics and proving that a sustainable process can also be quantified.

## 3. Methods and Materials

All reagents were used as received, purchased from Sigma-Aldrich (Munich, Germany). Solvents were purified using standard methods and freshly distilled under dinitrogen prior to use.

The chloro C-scorpionate iron(II) complex [FeCl_2_{κ^3^-HC(pz)_3_}] (pz, pyrazolyl) was prepared and characterized according to a published method [14].

The synthesized core-shell particles, before and after functionalization with [FeCl_2_{κ^3^-HC(pz)_3_}], were characterized by using scanning electron microscopy (SEM) and energy dispersive spectroscopy (EDS). Morphology of the core shells and the catalyst were characterized using a scanning electron microscope (JEOL 7001F with Oxford light elements EDS detector and EBSD detector, JEOL, Tokyo, Japan). The iron content of the core-shell supported catalyst was analyzed by using inductively coupled plasma atomic emission spectroscopy (ICP-AES) carried out in an ICP-AES model Ultima (Horiba Jobin-Yvon, Kyoto, Japan) apparatus.

Gas chromatographic (GC) was made using a FISONS Instruments GC 8000 series gas chromatograph equipped with a flame ionization (FID) detector and a capillary column (DB-WAX, column length of 30 m; column internal diameter of 0.32 mm) and run by the software Jasco-Borwin v.1.50 (Jasco, Tokyo, Japan). Helium was the carrier gas and the temperature of injection was 240 °C. After injection, the temperature was maintained at 140 °C for 1 min, then raised by 10 °C/min until 220 °C was reached and conditions were held for 1 min at this temperature. All products were identified by comparison of their retention times with a known reference.

### 3.1. Synthesis of Magnetic Fe_3_O_4_/TiO_2_/[FeCl_2_{κ^3^-HC(pz)_3_}] Composite

The synthesis of Fe_3_O_4_/TiO_2_/[FeCl_2_{κ^3^-HC(pz)_3_}] composite was performed by following a very recently reported [26] procedure. Briefly, a mixture of two salts ((NH_4_)_2_Fe(SO_4_)_2_·6H_2_O and FeCl_3_) was dissolved in distilled water at 50 °C and stirred while a solution of NaOH 1 M was added, until the pH 10 was achieved. The magnetic Fe_3_O_4_ was collected (with a magnet, after cooled down to room temperature), washed with distilled water and ethanol and dried overnight at 80 °C. In a second step, to ethanol, the previously synthetized Fe_3_O_4_, Ti(IV) isopropoxide and distilled water were added, and the mixture was left for two hours under reflux. As in the previous step, after the solution cooled to room temperature, the magnetic Fe_3_O_4_/TiO_2_ was separated, washed and dried under vacuum overnight. Finally, the as-prepared Fe_3_O_4_/TiO_2_ and [FeCl_2_{κ^3^-HC(pz)_3_}] were dissolved in distilled water and stirred for 24 h. The desired composite was obtained by using filtration and washed with distilled water. In order to control the presence of chlorides in the solution and understand when the compound was washed enough, a silver nitrate solution was used. Finally, Fe_3_O_4_/TiO_2_/[FeCl_2_{κ^3^-HC(pz)_3_}] was washed with methanol and dried under vacuum overnight.

### 3.2. Catalytic Oxidation of Ethane to Acetic Acid

All the reactions were carried out in stainless steel autoclaves by reacting at a typical temperature of 50–100 °C and in a selected medium (3 mL/3 mL), ethane (3–20 atm), [FeCl_2_{κ^3^-HC(pz)_3_}], Fe_3_O_4_/TiO_2_/[FeCl_2_{κ^3^-HC(pz)_3_}] or FeCl_2_.H_2_O (0.02 to 0.08 mmol) and potassium peroxodisulfate (4.33 mmol) as an oxidant. The reaction mixture was stirred typically between 6 and 72 h using an oil bath. Afterwards, it was cooled, degassed, opened and the contents transferred to a Schlenk flask. Diethyl ether (9.0 mL) and 90 μL of cycloheptanone (GC internal standard) were added.

The recyclability of catalyst Fe_3_O_4_/TiO_2_/[FeCl_2_{κ^3^-HC(pz)_3_}] was investigated under the highest yield experimental reaction conditions. Each cycle was initiated after the preceding one by adding of new above-mentioned portions of all other reagents. After completion of each run, the products were separated for analysis (see above) and catalyst separated by a magnet, washed and dried overnight at 70 °C.

## 4. Conclusions

This is the first time that a C-scorpionate complex anchored onto a magnetic core-shell support, Fe_3_O_4_/TiO_2_/[FeCl_2_{κ^3^-HC(pz)_3_}], was used as a catalyst for the challenging oxidation of ethane to acetic acid. The use of the Fe_3_O_4_/TiO_2_/[FeCl_2_{κ^3^-HC(pz)_3_}] composite led to a highly selective catalytic method for the acetic acid synthesis, where the magnetic catalyst is easily recovered and reused. In view of the promising results achieved, also attested to by the determined green metrics, further optimization studies will be attempted to help with the design of a sustainable catalytic process for this industrially important reaction.

## Figures and Tables

**Table 1:** Catalytic performance of [FeCl_2_{κ^3^-HC(pz)_3_] in the one-pot oxidation of ethane to acetic acid ^a^.

Entry	Cat/mmol	T/°C	C_2_H_6_/atm	Time/h	Solvent	Yield ^b^/%	TON ^c^
1	0.02	80	3	20	TFA	6.8	6
2	0.04	80	3	20	TFA	13.6	6
3	0.02	80	3	20	ACN/H_2_O	4.7	4
4	0.04	80	3	6	ACN/H_2_O	4.9	2
5	0.04	80	3	20	ACN/H_2_O	10.1	4
6	0.04	80	3	72	ACN/H_2_O	12.3	5
7	0.04	80	3	20	H_2_O	4.6	2
8	0.04	80	3	20	ACN	0.0	0
9	0.04	80	5	20	ACN/H_2_O	10.3	7
10	0.04	50	3	20	ACN/H_2_O	5.2	2
11 ^d^	0.04	80	3	20	ACN/H_2_O	0.0	0
12	-	80	3	6	ACN/H_2_O	3.9	0
13 ^e^	0.04	80	3	20	ACN/H_2_O	4.2	2

^a^ Reaction conditions: 4.33 mmol K_2_S_2_O_8_, 6 mL of solvent. Selectivity for acetic acid ≥99% for all assays. ^b^ Yield (%) = (moles of acetic acid/100 moles of ethane). ^c^ Moles of acetic acid/mole of catalyst. ^d^ in the presence of 0.008 mol of pyrazine carboxylic acid (HPCA). ^e^ in the presence of 0.04 mmol iron chloride (FeCl_2_.4H_2_O).

**Table 2:** Catalytic performance of Fe_3_O_4_/TiO_2_/[FeCl_2_{κ^3^-HC(pz)_3_}] composite in the oxidation of ethane ^a^.

Entry	Cat/mmol	T/°C	C_2_H_6_/atm	Time/h	Solvent	Aditive	Yield ^b^/%	TON ^c^
						mmol		
1	0.02	80	3	20	TFA	-	3.0	3
2	0.04	80	3	20	TFA	-	3.3	1
3	0.04	80	3	6	ACN/H_2_O	-	2.5	1
4	0.04	80	3	20	ACN/H_2_O	-	29.1	12
5	0.02	80	3	20	ACN/H_2_O	-	21.3	18
6	0.02	80	3	72	ACN/H_2_O	-	25.6	21
7 ^d^	0.02	80	3	20	ACN/H_2_O	8	2.7	2
8 ^e^	0.02	80	3	20	ACN/H_2_O	8	2.2	2
9	0.02	50	3	20	ACN/H_2_O	-	18.3	15
10	0.02	100	3	20	ACN/H_2_O	-	21.5	18
11	0.02	80	5	20	ACN/H_2_O	-	16.5	23
12	0.02	80	20	20	ACN/H_2_O	-	13.7	75
13	0.02	80	3	20	ACN	-	0.0	0
14	0.02	80	3	20	H_2_O	-	5.9	5
15	0.00	80	3	20	ACN/H_2_O	-	0.0	0
16 ^f^	0.08	80	3	20	ACN/H_2_O	-	6.7	6
17 ^f^	0.02	80	3	20	ACN/H_2_O	-	0.6	0
18 ^g^	0.02	80	3	20	ACN/H_2_O	-	0.0	0

^a^ Reaction conditions: 4.33 mmol K_2_S_2_O_8_, 6 mL of solvent. Cat is the quantity in mmol of [FeCl_2_{κ^3^-HC(pz)_3_}] present. Selectivity for acetic acid ≥ 99% for all assays. ^b^ Yield (%) = (moles of acetic acid/100 moles of ethane). ^c^ Moles of acetic acid/mole of catalyst. ^d^ 0.008 mmol of pyrazine carboxylic acid (HPCA); ^e^ 0.008 mmol of sodium hydroxide; ^f^ Fe_3_O_4_/TiO_2_/FeCl_2._2H_2_O. ^g^ Fe_3_O_4_/TiO_2_ core-shell.

**Table 3:** Green chemistry metrics for the oxidation of ethane to acetic acid catalyzed by [FeCl_2_{κ^3^-HC(pz)_3_}] or Fe_3_O_4_/TiO_2_/[FeCl_2_{κ^3^-HC(pz)_3_}].

Entry	Solvent	Catalyst	E-Factor ^a^	AE/% ^b^	AU/% ^c^	f ^d^
1	H_2_O/ACN	[FeCl_2_{κ^3^-HC(pz)_3_}]	0.5	20.0	100	108.4
2	TFA		2.7			702.3
3	H_2_O/ACN	Fe3O4/TiO2/ [FeCl2{κ3-HC(pz)3}]	0.7	20.0	100	122.1
4	TFA		1.9			570.3

^a^ Environmental factor (E-factor) [41] = total weight (in kg) of all waste generated in an industrial process per kilogram of product; ^b^ Atom Economy (AE) = the conversion efficiency of a chemical process in terms of all atoms involved and the desired products produced [42] ^c^ Reaction Mass Efficiency (RME) [41] = measures the efficiency with which reactant mass ends up in the desired product; ^c^ Atom Utilization (AU) = is the ratio of the molecular weight of the desired product to the sum total of all the materials (excluding solvents) used; ^d^ Solvent and Catalyst Environmental Impact Parameter (f) = the ratio of the quantity of all materials used to the quantity of materials produced.
